# Supplementary material for: Increased risk of developing dental diseases in patients with primary Sjögren’s syndrome—A secondary cohort analysis of population-based claims data
Source: PLoS One. 2020 Sep 18;15(9):e0239442. doi: 10.1371/journal.pone.0239442 (PMC7500664; doi:10.1371/journal.pone.0239442)
Supplement: Attachment — Submitted filename: Response to Reviewers Comments.doc [file pone.0239442.s001.doc]

**Manuscript ID: PONE-D-20-05093**

Title: Increased risk of developing dental diseases in patients with primary Sjögren’s syndrome– a secondary cohort analysis of population-based claims data

-------------------------------------------------------------------------------------------------

Journal requirement comment #1:

Please ensure that your manuscript meets PLOS ONE's style requirements, including those for file naming.

**Response to journal requirement comment #1:**

We have checked our manuscript to ensure it meets PLOS ONE’s style requirement.

-------------------------------------------------------------------------------------------------

Journal requirement comment #2:

We noticed you have some minor occurrence(s) of overlapping text with the following previous publication(s), which needs to be addressed:

https://doi.org/10.1007/s10067-019-04705-z

In your revision ensure you cite all your sources (including your own works), and quote or rephrase any duplicated text outside the Methods section. Further consideration is dependent on these concerns being addressed.

**Response to journal requirement comment #2:**

We have revised our manuscript, particularly the Introduction and Methods section to reduce the amount of overlapping text.

-------------------------------------------------------------------------------------------------

Journal requirement comment #3:

We note that you have indicated that data from this study are available upon request. PLOS only allows data to be available upon request if there are legal or ethical restrictions on sharing data publicly. For information on unacceptable data access restrictions, please see http://journals.plos.org/plosone/s/data-availability#loc-unacceptable-data-access-restrictions.

**Response to journal requirement comment #3:**

In the Data Availability statement, we indicated that “No – some restrictions will apply”. And, we indicated that “Due to legal restrictions imposed by the government of Taiwan in relation to the “Personal Information Protection Act”, data cannot be made publicly available. Requests for data can be sent as a formal application to the Health and Welfare Data Science Center, Department of Statistics, Ministry of Health and Welfare, Taiwan (http://dep.mohw.gov.tw/DOS/np-2497-113.html).”

-------------------------------------------------------------------------------------------------

**Reviewer #1 comment #1:**

As the aim of your study is to objectify side effects of pSS on oral health, you need to describe more precisely the salivary role : -buffer power

-physical obstacle

-hydration, lubrication

-Monitoring of oral microbiota,

-Tank of ions and minérals salts,

-Healing with EGF and VEGF.

You also need to emphasize on the prevention's problematic as it's difficult to provide a good oral restauration with removable denture when there is a lack of saliva.

**Response to Reviewer #1 comment #1:**

We agree with the reviewer. We have added the following description in the Introduction section: “The main functions of saliva include balancing the oral microbiota, providing lubrication and protection of the oral mucosa and tongue, facilitating carbohydrate digestion in the mouth, providing acid-base buffering and mineral salts, acting as a physical obstacle, and involving in wound healing mediated by epidermal growth factor (EGF) and vascular endothelial growth factor (VEGF) [4].

-------------------------------------------------------------------------------------------------

**Reviewer #1 comment #2:**

By the end, as clinical expression of xerostomia should be different between two patients who suffer of pSS, there is a missing data, you need to objective the clinical severity of dry mouth.

**Response to Reviewer #1 comment #2:**

Due to the inherent limitation of the NHIRD, information on the clinical severity of dry mouth was not available. However, it is known that the severity of xerostomia can increase with a longer duration of pSS. Therefore, we analyzed the incidence of dental diseases with the duration of the pSS diagnosis. We found that the IRRs are similar for dental caries, pulpitis, gingivitis, periodontitis, oral ulceration, and stomatitis in patients with pSS regardless of whether the disease duration was less than or equal to 5 years or longer than 5 years. The development of these dental diseases is highly complex. Although xerostomia may be an important factor, other physical, biological, environmental, behavioral, and lifestyle factors can also play critical roles in the pathogenesis of different dental diseases.

We have revised the relevant text in the Results section and the tables.

-------------------------------------------------------------------------------------------------

-------------------------------------------------------------------------------------------------

**Reviewer #2 comment #1:**

No correction for multiple comparisons has been performed.

**Response to Reviewer #2 comment #1:**

We appreciate the reviewer’s comment. We did not perform adjustment for multiple comparisons for two reasons. First, most of the results had a p value of < 0.001, and therefore will not materially affect our conclusions with or without adjustment. Second, our comparisons were not post-hoc comparisons performed after a significant overall comparison was observed, such as the case of ANOVA. We hope that the reviewer will agree with our approach.

-------------------------------------------------------------------------------------------------

**Reviewer #2 comment #2:**

As the authors address risk factor of dental disease, they may address specifically the effect of treatment for Sjögren’s syndrome (e.g. did MTX-treated patients had more stomatitis? Does treatment affect the risk at all?). They may further address the effect of disease duration, since diagnosis and the one of extraglandular disease on the risk for dental disease.

**Response to Reviewer #2 comment #2:**

As for the possible effect of treatment on the development of dental diseases, we only selected patients with primary Sjögren’s syndrome (pSS). MTX is rarely use in patients with pSS (less than 5%; according to Hsu et al. Arthritis Res Ther. 2019; 21: 251). Therefore, we did not further analyze the effect of MTX in our study. The common treatment for pSS in Taiwan is pilocarpine (28%). It was not found offer any protective on dental caries, periodontitis, or oral candidiasis in patients with pSS as shown in a previous study using the same NHIRD database (Hsu et al. Arthritis Res Ther. 2019; 21: 251).

It is known that the salivary flow is significantly reduced with a longer duration of pSS. Nevertheless, we found that the IRRs were similar for pulpitis, gingivitis, periodontitis, oral ulceration, and stomatitis in patients with pSS, compared with the comparison cohort regardless of whether the disease duration was less than or equal to 5 years or greater than 5 years. In fact, the IRR was not significantly elevated in dental caries in pSS patients with a disease duration greater than 5 years. The development of these dental diseases is highly complex. Although xerostomia may be an important factor, other physical, biological, environmental, behavioral, and lifestyle factors can also play critical roles in the pathogenesis of different dental diseases. We have revised the tables and the relevant part of the Discussion.

-------------------------------------------------------------------------------------------------

-------------------------------------------------------------------------------------------------
